# ^15^N and ^31^P NMR Studies of Cyano[(Trialkyl/Triaryl)Phosphine]Gold(I) Complexes R_3_PAu^13^C^15^N

**DOI:** 10.1155/MBD.1994.514

**Published:** 1994

**Authors:** M. Naseem Akhtar, Anvarhusein A. Isab, Abdul Rahman Al-Arfaj, Mohammed I. M. Wazeer

**Affiliations:** 1 Department of Chemistry King Fahd University of Petroleum and Minerals Dharan 31261 Saudi Arabia


					5N AND 31p NMR STUDIES OF
CYANO[(TRIALKYL/TRIARYL)PHOSPHINE]GOLD(1)
COMPLEXES R3PAu13C15N
M. Naseem Akhtar, Anvarhusein A. Isab,
Abdul Rahman AI-Arfaj and Mohammed I. M. Wazeer
Department of Chemistry, King Fahd University of Petroleum and Minerals,
Dharan 31261, Saudi Arabia
The ligand scrambling reaction of R3PAuC15N to form (R3P)2Au+ and Au(C15N)2" has
been studied for R=Me, Et, i-Pr, and Ph. The reactions are studied by 15N and 31p NMR
spectroscopy. Two 31p NMR resonances were observed for R3PAuCN and (R3P)2Au+
species. However, for the 15N NMR, only averaged resonances were observed for
R3PAuC15N and Au(C15N)2" species except for Et3PAuC15N, where two resonances
were detected.The R3PAu13C15N complexes have also been prepared where R= Me, Et,
and Ph. Analysis of 31p NMR of R3PAu13C15N (where R=Me, Et and Ph yielded
2j(31p.13C) values as 120.71, 122.18 and 124.57 Hz and 3j (31p.15N) values as 3.62,
2.93 and 4.02 Hz respectively. The equilibrium constant (Keq) was measured to be 0.14
between -60C to -40C for Ph3PAuCN and zG# was determined by line-shape analysis of
31 p NMR peaks to be 39.7 + 0.5 kJ/mol at 273 K.
514

				

